# Predictive values of the selected inflammatory index in elderly patients with papillary thyroid cancer

**DOI:** 10.1186/s12967-018-1636-y

**Published:** 2018-09-21

**Authors:** Weiheng Wen, Peili Wu, Jitong Li, He Wang, Jia Sun, Hong Chen

**Affiliations:** 1 Department of Endocrinology, Zhujiang Hospital, Southern Medical University, Guangzhou, 510282 People’s Republic of China; 2 Department of Thoracic Surgery, Nanfang Hospital, Southern Medical University, Guangzhou, 510282 People’s Republic of China

**Keywords:** Papillary thyroid cancer, Tumor-node-metastasis staging system, Inflammatory index, Lymphocyte-to-monocyte ratio, Prognosis

## Abstract

**Background:**

The American Joint Committee on Cancer has recently revised the tumor-node-metastasis (TNM) staging system on thyroid cancer, which illustrated that the cut-off age for predicting mortality has elevated from 45 to 55 years old. We aimed to investigate the inflammation index based on hematological parameters to predict the clinical characteristics of elderly papillary thyroid cancer (PTC) patients with an inferior prognosis.

**Methods:**

We retrospectively analyzed 558 patients newly diagnosed with PTC from January 2013 to December 2017, and 82 out of the 558 patients were over 55 years old. Receiver operating characteristic (ROC) study and univariate and multivariate logistic analysis was conducted to evaluate the diagnostic value of these preoperative inflammation indexes in PTC patients ≥ 55 years of age.

**Results:**

Elevated neutrophils were prognostic of bilaterality (area under the ROC curve (AUC) = 0.673, p = 0.023) and lymph node metastasis (AUC = 0.649, p = 0·020). Decreased mean platelet volume (MPV) and platelet distribution width (PDW) were prognostic of coexistence with Hashimoto’s thyroiditis (AUC = 0.736, p = 0.016; AUC = 0.721, p = 0.024). Elevated lymphocyte and lymphocyte-to-monocyte ratio (LMR) were prognostic of advanced TNM stage (AUC = 0.691, p = 0.029; AUC = 0.680, p = 0.040). Meanwhile, the logistic regression model further revealed that LMR ≥ 5.45 was an independent risk factor for the advanced TNM stage (odds ratio (OR) = 7.306, p = 0.036).

**Conclusions:**

The preoperative neutrophils, lymphocytes, MPV, PDW, LMR were all prognostic. More importantly, the increased in LMR independently contributed to the advanced TNM stage of PTC patients ≥ 55 years.

**Electronic supplementary material:**

The online version of this article (10.1186/s12967-018-1636-y) contains supplementary material, which is available to authorized users.

## Background

Papillary thyroid cancer (PTC) is the most epidemic type of thyroid cancers, accounting for approximately 80% of all diagnosed thyroid cancers [1]. Over the past decade, PTC has maintained its prevalence in some regions of the world, leading to a higher incidence of PTC than that of other cancers. However, the prognosis of PTC, which is greater than 90% in 10-year survival postoperatively, is much better than the vast majority of other malignancies [1, 2]. However, patients with PTC still suffer unnecessary cost and anxiety, which is the result of overtreatment [3]. The American Joint Committee on Cancer (AJCC) has recently revised the tumor-node-metastasis (TNM) staging system on thyroid cancer, revealing a looser standard in stage II, which reduces instances of overtreatment when following the clinical management guidelines. Regarding the PTC staging system changes, it should be noted that lymph node metastasis is no longer an indicator of advanced stage, and the cut-off age for predicting mortality has elevated from 45 to 55 years old [4]. These modifications have been supported by several studies [5–7]. With the new staging, PTC patients under 55 years of age, are all in the early stage (I–II). Therefore, these patients do not need to accept radical, unnecessary therapy, while PTC patients over 55 years of age need additional information to determine whether further treatment is needed.

Cancer-related inflammation is well-known for its vital role in carcinogenesis and the progression of neoplastic diseases, including PTC [8]. Since the routine blood test could reflect inflammatory response, at least to some extent, researchers have focused on the role of hematological parameters that act as predictive or prognostic indicators of carcinoma. Neutrophil count, lymphocyte count, the neutrophil-to-lymphocyte ratio (NLR), the lymphocyte-to-monocyte ratio (LMR), mean platelet volume (MPV) and platelet distribution width (PDW) have been well-studied in many malignancies [9–11]. Nevertheless, these potentially related carcinoma markers are less involved in PTC, and even among those studies, the relationship between hematological index and metastasis or stage of PTC are inconsistent, both of which remain in dispute [12, 13]. More importantly, as the PTC staging system has been changed, previous studies involving PTC stage may be less effective for evaluating its relationship with current hematological parameters. Therefore, this study set out to investigate the role of hematological indexes in predicting clinical characteristics and outcomes of PTC, particularly in elderly patients (≥ 55 years old).

## Methods

From January 2013 to December 2017, a total of 558 PTC patients, who were diagnosed by pathology results after initial thyroidectomy, at the affiliated hospitals of Southern Medical University, Nanfang Hospital were enrolled in this study. Patients who had accepted initial surgical operation at another hospital, patients with tumors that were considered to be unresectable, patients who had other malignancies or had accepted cancer therapy within the past year, and patients with incomplete inspection data were excluded. We retrospectively collected data, including age, gender, as well as clinicopathological data regarding tumor diameter, presence or absence of tumor bilaterality, site of lymph node metastasis, presence or absence of Hashimoto’s thyroiditis. The primary tumor size and its extent (T), lymph node involvement (N), and distant metastases (M) of PTC were based on the new AJCC TNM scoring (Additional file 1: Table S1). And then we reclassified the stage of PTC patients. In short, the cut-off age at diagnosis has elevated from 45 to 55 years old. PTC patients over 55 years old that satisfied T4a/T4b status or M1 status are classified as advanced stage (III-IV). Notably, N1 category does no longer automatically mandate stage III or IV (Additional file 2: Table S2). These modifications contribute to downstaging of a considerable number of patients and thus improve the prediction of survival in PTC patients. In addition, the inflammatory index, such as complete neutrophil and lymphocyte counts, were also collected from preoperative routine blood test. The value of NLR was calculated by dividing the complete neutrophil count by the complete lymphocyte count, whereas the value of LMR was calculated by dividing the complete lymphocyte count by the complete monocyte count. This cohort study was performed in accordance with the Declaration of Helsinki to protect the patients’ individual information.

Continuous variables were expressed as mean ± standard deviation (SD), while categorical variables were presented as percentages. For comparisons, the *t* test or Mann–Whitney U test was performed, depending on the normality of the data distribution. The Chi squared test was used for categorical variables. The evaluation of optimum cut-off values for hematological parameters was performed using a receiver operating characteristics (ROC) curve. The univariate and multivariate analysis was conducted to assess the prognostic factors using the logistic regression model. Differences were considered statistically significant at p < 0.05, and all p values were based on 2-sided tests. The SPSS version 22.0 software was used for statistical calculations.

## Results

### Comparisons between young group and elder group

A total of 558 patients who accepted surgical therapy for previously untreated PTC were enrolled in this retrospective analysis. Of these, 82 patients were older than 55 years (including 55 years), whereas 476 patients were less than 55 years of age. As shown in Table 1, elderly patients with PTC were less likely to experience lymph node metastasis (51.2% vs 68·2%; p = 0.003) and Hashimoto’s thyroiditis (12.2% vs 24.0%; p = 0.018). No significant differences were detected between two groups concerning tumor size and bilaterality.

**Table 1:** Demographic and clinical characteristics of patients with PTC

	Total	Age < 55 years	Age ≥ 55 years	p
Total	558 (100%)	476 (85.3%)	82 (14.7%)	
Age (years)	39.29 ± 12.06	35.68 ± 8.79	60.28 ± 4.96	0.000*
Gender				
Female	378 (67.7%)	322 (67.6%)	56 (68.3%)	0.908
Male	180 (32.3%)	154 (32.4%)	26 (31.7%)	
Largest tumor size (cm)	1.54 ± 1.24	1.53 ± 1.16	1.56 ± 1.66	0.859
Bilaterality				
Yes	136 (24.4%)	117 (24.6%)	19 (23.3%)	0.784
No	422 (75.6%)	359 (75.4%)	63 (76.8%)	
Lymph node metastasis				
Yes	366 (65.6%)	324 (68.1%)	42 (51.2%)	0.003*
No	192 (34.4%)	152 (31.9%)	40 (48.8%)	
Coexistence with Hashimoto’s thyroiditis				
Yes	131 (23.5%)	121 (25.4%)	10 (12.2%)	0.009*
No	427 (76.5%)	355 (74.6%)	72 (87.8%)	

Data are expressed as mean ± standard deviation or number (%)

The significance level was set at p < 0.05

* Significant difference between the two groups

### Analysis of PTC in elderly patients

**Fig. 1:**
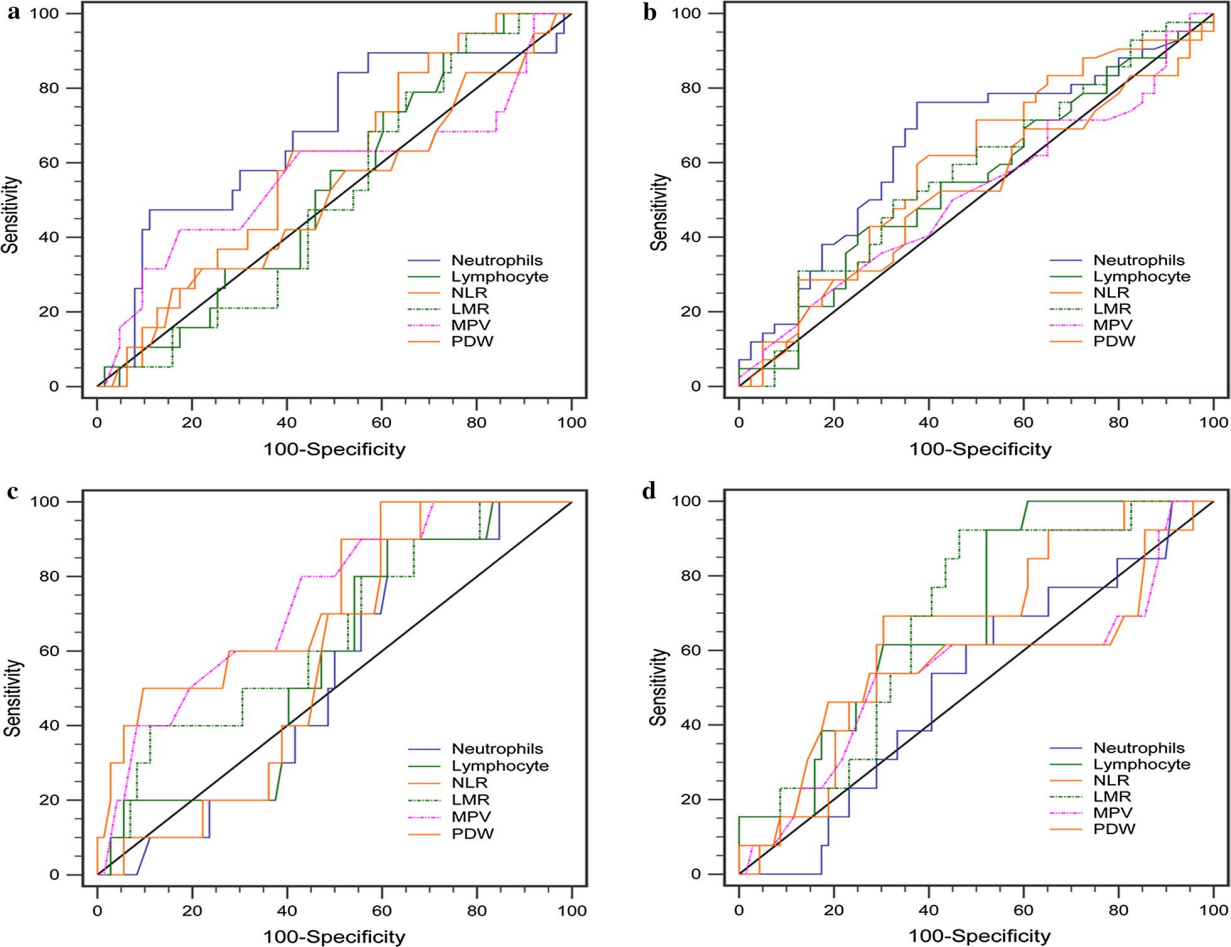
ROC for determination of predictive ability of hematological parameters in elderly patients with PTC to: **a** bilaterality, **b** lymph node metastasis, **c** coexistence with Hashimoto’s thyroiditis, and **d** TNM stage, respectively. Abbreviations: ROC, receiver operating curve; NLR, neutrophil-to-lymphocyte ratio; LMR, lymphocyte-to-monocyte ratio; MPV, and mean platelet volume; PDW, platelet distribution width

ROC curve analysis was conducted to predict bilaterality, lymph node metastasis, coexistence with Hashimoto’s thyroiditis, and TNM stage, respectively (Fig. 1), and the area under the ROC curve (AUC) values were also evaluated in our study (Table 2). The AUC of neutrophils for predicting bilaterality was 0.673 (95% confidence interval (CI) 0.529–0.818; p = 0.023). The best cut-off value was 4.93 G/L, with a sensitivity of 47.4% and a specificity of 88.9%. The AUC for lymphocytes, NLR, LMR, MPV, and PDW to predict bilaterality were 0.537 (p = 0.629), 0.601 (p = 0.185), 0·505 (p = 0.943), 0.569 (p = 0.362), and 0.509 (p = 0.904) correspondingly.

**Table 2:** AUC of hematological parameters of clinicopathologic characteristics in elderly patients (≥ 55 years) with PTC

Prognostic factor	AUC	95% CI	p
Bilaterality			
Neutrophils	0.673	0.529–0.818	0.023*
Lymphocyte	0.537	0.401–0.673	0.629
NLR	0.601	0.466–0.735	0.185
LMR	0.505	0.372–0.639	0.943
MPV	0.569	0.402–0.737	0.362
PDW	0.509	0.357–0.661	0.904
Lymph node metastasis			
Neutrophils	0.649	0.528–0.771	0.020*
Lymphocyte	0.550	0.424–0.676	0.436
NLR	0.603	0.479–0.726	0.109
LMR	0.575	0.450–0.700	0.242
MPV	0.516	0.390–0.642	0.802
PDW	0.519	0.393–0.646	0.763
Coexistence with Hashimoto’s thyroiditis			
Neutrophils	0.528	0.377–0.680	0.771
Lymphocyte	0.575	0.411–0.739	0.444
NLR	0.594	0.460–0.727	0.339
LMR	0.640	0.462–0.818	0.152
MPV	0.736	0.584–0.888	0.016*
PDW	0.721	0.548–0.893	0.024*
Advanced TNM stage			
Neutrophils	0.515	0.358–0.672	0.864
Lymphocyte	0.691	0.558–0.825	0.029*
NLR	0.653	0.505–0.801	0.081
LMR	0.680	0.545–0.815	0.040*
MPV	0.545	0.352–0.737	0.612
PDW	0.564	0.366–0.763	0.465

AUC, area under the curve of ROC; NLR, neutrophil-to-lymphocyte ratio; LMR, lymphocyte-to-monocyte ratio; MPV, mean platelet volume; PDW, platelet distribution width

The significance level was set at P < 0.05

* Significant difference

With regard to prediction of lymph node metastasis, the AUC of neutrophils was 0.649 (95% CI 0.528–0.771; p = 0.020). The ideal cut off value was 3.45 G/L, with a sensitivity of 76.2% and a specificity of 62.5%. However, lymphocyte, NLR, LMR, MPV, PDW showed little predictive capacity, with AUC value of 0.550 (p = 0.436), 0.603 (p = 0.109), 0.575 (p = 0.242), MPV (p = 0.802), and 0.519 (p = 0.763), respectively.

In case of coexistence with Hashimoto’s thyroiditis, the AUC value of MPV was 0.736 (95% CI 0.584–0.888; p = 0·016). According to the cut-off values, MPV>10.35 fl had an ideal diagnostic accuracy, with a sensitivity of 56.9% and a specificity of 80%. Similarly, the AUC value of PDW to predict coexistence with Hashimoto’s thyroiditis was 0.721 (95% CI 0.548–0.893; p = 0.024). The optimal cut-off value was 10.55, with a sensitivity of 90.3% and a specificity of 50%. AUC values for neutrophils, lymphocytes, NLR, and LMR to predict coexistence with Hashimoto’s thyroiditis were respectively 0. 528 (p = 0.771), 0.575 (p = 0.444), 0.594 (p = 0.339), and 0.640 (p = 0.152).

Additionally, a ROC curve to predict advanced TNM stage was plotted. It demonstrated that the AUC value of lymphocytes to predict advanced TNM stage was 0.691 (95% CI 0.558–0.825; p = 0.029). The optimal cut-off value was 1.70 G/L, with a sensitivity of 92.3% and a specificity of 47.8%. Equivalently, the AUC value of LMR was 0.695 (95% CI 0.556–0.835; p = 0.031). A cut-off value of 5.45 was found to be the optimal diagnosis potential, with a sensitivity of 91.7% and a specificity of 57.1%. However, the AUC values of neutrophils, NLR, MPV, and PDW to predict advanced TNM stage were correspondingly 0.515 (p = 0.864), 0.653 (p = 0.081), 0.545 (p = 0.612), and 0.564 (p = 0.465). ROC curve analysis of clinical characteristics in PTC patients less than 55 years old has also been performed (Additional file 3: Table S3).

Univariate and multivariate logistic analyses were conducted to identify further whether the lymphocyte count and LMR values were independently correlated to the advanced TNM stage (Table 3). Using the lymphocyte count and LMR cut-off points calculated by ROC curve analysis, the results showed that LMR ≥ 5.45 was an independent risk factor (odds ratio (OR) = 7.306, p = 0.036) for the advanced TNM stage in PTC patients.

**Table 3:** Univariate and multivariate analysis for advanced TNM stage

	Univariate analysis		Multivariate analysis	
	OR (95% CI)	P	OR (95% CI)	p
Age ≥ 55 (years)	1.097 (0.984–1.223)	0.094	1.152 (1.001–1.326)	0.049*
Gender, male/female	1.667 (0.418–6.651)	0.469	–	–
Largest tumor size (cm)	0.977 (0.665–1.436)	0.906	–	–
Bilaterality	0.994 (0.244–4.055)	0.993	–	–
Lymph node metastasis	1.647 (0.490–5.540)	0.420	–	–
Coexistence with Hashimoto’s thyroiditis	1.386 (0.259–7.418)	0.703	–	–
LMR ≥ 5.45	7.150 (1.473–34.714)	0.015*	7.306 (1.135–47.043)	0.036*
Lymphocyte ≥ 1.70(G/L)	11.000 (1.355–89.288)	0.025*	–	

LMR, the lymphocyte-to-monocyte ratio

The significance level was set at p < 0.05

* Significant difference

## Discussion

Thyroid cancer is one of the most rapidly changing cancers of the past decade, and its staging system has undergone significant revisions in line with the eighth version of the AJCC Staging Guideline. Since the diagnosis of age is an independent risk factor and closely related to the prognosis of thyroid cancer, the cut-off age for predicting mortality has elevated from 45 to 55 years old [2, 4]. After the implementation of the new version, thyroid papillary carcinoma under 55 years of age is classified as early (I–II period) stage. Also, a large proportion of middle-stage patients will be downstage and will, therefore, receive more appropriate treatment to improve their prognosis and quality of life. More auxiliary diagnostic data are needed to evaluate the necessity of additional therapy for patients over 55 years of age (including 55 years).

Recently, the association between carcinoma and chronic inflammation has been well-studied [14]. Also, the blood parameters as the simple index of the inflammatory system, including lymphocyte count, neutrophil count, LMR, NLR, MPV, and PDW, have been widely used as the predictive factors in determining the prognosis of some types of cancers [9–11]. However, few studies have investigated this association with PTC, and the value of hematological indexes in determining the prognosis of PTC is still under debate [12]. Therefore, this study sought to assess the predictive value of inflammatory indexes in evaluating the prognostic clinical characteristics of PTC, especially in elderly patients.

Our study suggested for the first time that preoperative neutrophil count was positively associated with bilaterality and lymph node metastasis, and both MPV and PDW were negatively associated with coexistence with Hashimoto’s thyroiditis. Our work also considered both lymphocyte count and LMR as predictive risk factors for the advanced TNM stage in elderly PTC patients. Notably, the multivariate logistic analysis showed that LMR ≥ 5.45 (OR 7.306, p = 0.036)was defined as an independent prognostic parameter to predict advanced TNM stage.

As part of the host immune reaction, neutrophils exhibit diverse functions to dynamically regulate cancer-related processes. Among these, it has been demonstrated that tumor-induced neutrophils can facilitate tumor metastases via circulation [15]. This mechanism is also supported by various clinical retrospective analyses, including ours [10]. In our study, we found that an elevated neutrophil count was related to bilaterality and more LNM in elderly patients with PTC, and this result is similar to descriptions in other carcinomas. Of note, the rate of lymph node metastasis in young patients was higher than that in elderly patients (68.1% vs 51.2%, p = 0.003), which is consistent with the phenomenon that the impact of lymphatic metastasis on staging system has been weakened by AJCC, and was excluded officially in stratifying advanced stage of elderly PTC patients [4]. Hence, although preoperative neutrophils were found to be associated with LNM, its indication function in advanced stage disease seemed insignificant in elderly PTC patients according to our observations, and further prospective studies are required to confirm.

Similar to neutrophils, platelets play a pivotal role in cancer progression and metastasis. Emerging evidence suggests that platelets regulate neoangiogenesis, dissemination, and tumor cell growth [16]. However, platelet activation is closely by their size rather than their count. Platelet size can be evaluated by platelet volume parameters, such as MPV and PDW. MPV is an alternative index of platelet activation, and PDW demonstrates variation in platelet size, and both have been used to predict the prognosis of various cancers [11, 17]. In our study, MPV and PDW were found to coexist with Hashimoto’s thyroiditis in PTC patients. MPV ≤ 10.35 fl and PDW ≤ 10.55 fl were considered as indicators of coexistence with Hashimoto’s thyroiditis, respectively. Several studies have indicated that Hashimoto’s thyroiditis may have a protective effect on PTC patients [18, 19]. Also, our present study also found that young PTC patients were more likely suffer from Hashimoto’s thyroiditis than elderly PTC patients (12.2% vs 35.4%; p = 0.009), who is considered to be a cohort with an inferior prognosis. Taken together, lower MPV and PDW might be a protective indicator for elderly PTC patients.

Previous studies have suggested the predictor value of LMR for clinical outcome in various types of cancer. Accumulating data indicates that a high LMR could be used to predict features of a variety of malignancies, such as nasopharyngeal carcinoma, gastric cancer, hepatocellular carcinoma, breast cancer, and colorectal carcinoma [9, 20–22]. Contrary to expectation, our data showed that high preoperative LMR, as well as lymphocyte count, were linked with advanced TNM stage, which is inconsistent with most studies. Also, according to multivariate analysis, LMR ≥ 5.45 (OR = 7.306, p = 0.036) was identified as an independent risk factor for the advanced stage in elderly PTC patients.

Obviously, lymphocytes, as the fundamental components of the adaptive immune system, are associated with healthy tumor profile by hindering tumor cells proliferation, inhibiting migration, and destroying metastases [23]. Previous studies have also proven the positive effect of increasing lymphocyte count in achieving a better survival in patients with advanced cancer [10]. In addition, the function of monocytes in carcinoma has been a topic of debate as it affects tumor progression in two contradicting ways. Macrophages of the liver (Kupffer cells) destroy circulating cancer [24], while, inversely, macrophages also help to spread tumor cells through circulation [25]. Consequently, compared to reduced macrophages, an increased lymphocyte count takes a more predominant place in high LMR-related improved cancer-related survival [9, 26].

However, unlike other carcinomas, the intricate role of lymphocytes in PTC remains unclear, as autoimmune disease occurred more frequently in the thyroid gland than any other organ. It has been widely accepted that PTC has an immunological association with autoimmune disease because both disorders are described to appear synchronously [27]. However, interactions between them are unclear and require further evidence. Some studies insist that elevated lymphocytes facilitate aggressive tumor behavior and thus result in reduced survival, and these are in accordance with our finding that elevated lymphocyte counts reflected a higher stage of PTC [25, 28, 29]. A similar positive correlation trend is also found in medullary thyroid carcinoma [30]. Our data may offer evidence of the association between lymphocytes and adverse invasive behavior in PTC. However, to further confirm their relationship with PTC and expand these findings, the need for more profound investigations is highlighted.

Taken together, our analysis delivers the evidence that pretreatment peripheral indexes could function as inexpensive indicators of aggressive behavior and higher stage in elderly patients with PTC, and LMR acted as an independent predictive factor in an advanced stage. In spite of these findings, our observation should be cautiously interpreted due to several limitations of our study. Firstly, our study is limited due to the inherent nature of retrospective analysis itself, leading to selection biases and inaccurate data recording. Secondly, although patients with diseases that could influence leucocytes were excluded, the result of blood-circulating cell counts may be impacted by other unknown or undetectable factors, such as medication. Lastly, the cases enrolled in our study were relatively small-scale, which tends to weaken the power of the study. Further multicenter or prospective studies are warranted to validate our conclusion.

## Conclusions

The present study is the first work to characterize inflammatory index in elderly patients with papillary thyroid cancer under the latest AJCC Staging Guideline. We found that pretreatment peripheral indexes could function as inexpensive indicators of aggressive behavior and higher stage in elderly patients with PTC, which may provide additional information to determine whether further treatment is necessary. The preoperative neutrophils, lymphocytes, MPV, PDW, LMR were all prognostic. More importantly, with LMR ≥ 5.45 as a cut-off value, the increased in LMR independently contributed to the advanced TNM stage of PTC patients ≥ 55 years, which inconsistent with most studies on other cancers rather than medullary thyroid carcinoma. This could be explained by the fact that the role of immune response in thyroid carcinoma remains complicated. Therefore, further basic researches and multicenter prospective studies on PTC are warranted to validate our conclusion.

## Additional files

**Additional file 1: Table S1.** AJCC TNM seventh and eighth edition: category.

**Additional file 2: Table S2.** AJCC TNM seventh and eighth edition: stage.

**Additional file 3: Table S3.** AUC of hematological parameters of clinicopathologic characteristics in young patients (<55 years) with PTC.

## Abbreviations

PTC: papillary thyroid cancer
HT: Hashimoto’s thyroiditis
TNM: tumor-node-metastasis
AJCC: American Joint Committee on Cancer
NLR: neutrophil-to-lymphocyte ratio
LMR: lymphocyte-to-monocyte ratio
MPV: and mean platelet volume
PDW: platelet distribution width
ROC: receiver operating characteristic

## References

[1] McLeod DS, Sawka AM, Cooper DS (2013). Controversies in primary treatment of low-risk papillary thyroid cancer. Lancet.

[2] Davies L, Welch HG (2014). Current thyroid cancer trends in the United States. JAMA Otolaryngol Head Neck Surg..

[3] Jegerlehner S, Bulliard JL, Aujesky D, Rodondi N, Germann S, Konzelmann I (2017). Overdiagnosis and overtreatment of thyroid cancer: a population-based temporal trend study. PLoS ONE.

[4] Lamartina L, Grani G, Arvat E, Nervo A, Zatelli MC, Rossi R (2017). 8th edition of AJCC/TNM staging system of thyroid cancer: what to expect. Endocr Relat Cancer..

[5] Kim SJ, Myong JP, Suh H, Lee KE, Youn YK (2015). Optimal cutoff age for predicting mortality associated with differentiated thyroid cancer. PLoS ONE.

[6] Nixon IJ, Kuk D, Wreesmann V, Morris L, Palmer FL, Ganly I (2016). Defining a valid age cutoff in staging of well-differentiated thyroid cancer. Ann Surg Oncol.

[7] Nixon IJ, Wang LY, Palmer FL, Tuttle RM, Shaha AR, Shah JP (2014). The impact of nodal status on outcome in older patients with papillary thyroid cancer. Surgery..

[8] Balkwill F, Mantovani A (2001). Inflammation and cancer: back to Virchow?. Lancet.

[9] Chan JC, Chan DL, Diakos CI, Engel A, Pavlakis N, Gill A (2017). The lymphocyte-to-monocyte ratio is a superior predictor of overall survival in comparison to established biomarkers of resectable colorectal cancer. Ann Surg.

[10] He JR, Shen GP, Ren ZF, Qin H, Cui C, Zhang Y (2012). Pretreatment levels of peripheral neutrophils and lymphocytes as independent prognostic factors in patients with nasopharyngeal carcinoma. Head Neck.

[11] Cheng S, Han F, Wang Y, Xu Y, Qu T, Ju Y (2017). The red distribution width and the platelet distribution width as prognostic predictors in gastric cancer. Bmc Gastroenterol..

[12] Lang BH, Ng CP, Au KB, Wong KP, Wong KK, Wan KY (2014). Does preoperative neutrophil lymphocyte ratio predict risk of recurrence and occult central nodal metastasis in papillary thyroid carcinoma?. World J Surg.

[13] Kim JY, Park T, Jeong SH, Jeong CY, Ju YT, Lee YJ (2014). Prognostic importance of baseline neutrophil to lymphocyte ratio in patients with advanced papillary thyroid carcinomas. Endocrine.

[14] Elinav E, Nowarski R, Thaiss CA, Hu B, Jin C, Flavell RA (2013). Inflammation-induced cancer: crosstalk between tumours, immune cells and microorganisms. Nat Rev Cancer.

[15] Coffelt SB, Wellenstein MD, de Visser KE (2016). Neutrophils in cancer: neutral no more. Nat Rev Cancer.

[16] Jiang L, Luan Y, Miao X, Sun C, Li K, Huang Z (2017). Platelet releasate promotes breast cancer growth and angiogenesis via VEGF-integrin cooperative signalling. Br J Cancer.

[17] Tanriverdi O, Menekse S, Teker F, Oktay E, Nur PK, Gunaldi M (2016). The mean platelet volume may predict the development of isolated bone metastases in patients with breast cancer: a retrospective study of the Young Researchers Committee of the Turkish Oncology Group (TOG). J Buon..

[18] Dvorkin S, Robenshtok E, Hirsch D, Strenov Y, Shimon I, Benbassat CA (2013). Differentiated thyroid cancer is associated with less aggressive disease and better outcome in patients with coexisting Hashimotos thyroiditis. J Clin Endocrinol Metab.

[19] Lee JH, Kim Y, Choi JW, Kim YS (2013). The association between papillary thyroid carcinoma and histologically proven Hashimoto’s thyroiditis: a meta-analysis. Eur J Endocrinol.

[20] Zhou X, Du Y, Xu J, Huang Z, Qiu T, Wang X (2014). The preoperative lymphocyte to monocyte ratio predicts clinical outcomes in patients with stage II/III gastric cancer. Tumour Biol.

[21] Song W, Tian C, Wang K, Zhang RJ, Zou SB (2017). The pretreatment lymphocyte to monocyte ratio predicts clinical outcome for patients with hepatocellular carcinoma: a meta-analysis. Sci Rep..

[22] He J, Lv P, Yang X, Chen Y, Liu C, Qiu X (2016). Pretreatment lymphocyte to monocyte ratio as a predictor of prognosis in patients with early-stage triple-negative breast cancer. Tumour Biol.

[23] Bastid J, Bonnefoy N, Eliaou JF, Bensussan A (2014). Lymphocyte-derived interleukin-17A adds another brick in the wall of inflammation-induced breast carcinogenesis. Oncoimmunology..

[24] Tavares AJ, Poon W, Zhang YN, Dai Q, Besla R, Ding D (2017). Effect of removing Kupffer cells on nanoparticle tumor delivery. Proc Natl Acad Sci USA..

[25] Qing W, Fang WY, Ye L, Shen LY, Zhang XF, Fei XC (2012). Density of tumor-associated macrophages correlates with lymph node metastasis in papillary thyroid carcinoma. Thyroid..

[26] Cunha LL, Morari EC, Guihen AC, Razolli D, Gerhard R, Nonogaki S (2012). Infiltration of a mixture of immune cells may be related to good prognosis in patients with differentiated thyroid carcinoma. Clin Endocrinol (Oxf).

[27] Ehlers M, Schott M (2014). Hashimoto’s thyroiditis and papillary thyroid cancer: are they immunologically linked?. Trends Endocrinol Metab.

[28] French JD, Kotnis GR, Said S, Raeburn CD, McIntyre RJ, Klopper JP (2012). Programmed death-1 + T cells and regulatory T cells are enriched in tumor-involved lymph nodes and associated with aggressive features in papillary thyroid cancer. J Clin Endocrinol Metab.

[29] Yu H, Huang X, Liu X, Jin H, Zhang G, Zhang Q (2013). Regulatory T cells and plasmacytoid dendritic cells contribute to the immune escape of papillary thyroid cancer coexisting with multinodular non-toxic goiter. Endocrine.

[30] Jiang K, Lei J, Li C, Shu K, Li W, Zhang Y (2017). Comparison of the prognostic values of selected inflammation based scores in patients with medullary thyroid carcinoma: a pilot study. J Surg Oncol.

