# Supplementary material for: Predictive values of the selected inflammatory index in elderly patients with papillary thyroid cancer
Source: J Transl Med. 2018 Sep 21;16:261. doi: 10.1186/s12967-018-1636-y (PMC6151008; doi:10.1186/s12967-018-1636-y)
Supplement: Supplementary file 1 — Additional file 1: Table S1. AJCC TNM seventh and eighth edition: category. [file 12967_2018_1636_MOESM1_ESM.docx]

|  | **7^th^ edition** | | **8^th^ edition** | |
| --- | --- | --- | --- | --- |
| **Tumor** | **T0** | no evidence of primary tumor | The same. | |
|  | **T1a** | tumor≤1 cm AND intrathyroidal | The same. | |
|  | **T1b** | tumor∈ ( 1,2cm] AND intrathyroidal | The same. | |
|  | **T2** | tumor∈ ( 2,4cm] AND intrathyroidal | The same. | |
|  | **T3** | tumor＞4cm AND intrathyroidal  OR  minimal extrathyroid extension  (perithyroidal soft tissues or sternothyroid muscle) | **T3a** | tumor＞4cm AND intrathyroidal |
|  |  |  | **T3b** | gross extrathyroidal extension invading only strap muscles (sternohyoid, sternothyroid,  thyrohyoid, omohyoid muscles) |
|  | **T4a** | gross extrathyroidal extension  (subcutaneous soft tissues, larynx, trachea, esophagus, or recurrent laryngeal nerve) | The same. | |
|  | **T4b** | gross extrathyroidal extension  (prevertebral fascia or encasing the carotid artery or mediastinal vessels) | The same. | |
| **Node** | **Nx** | regional lymph nodes cannot be assessed | The same. | |
|  | **N0** | no evidence of regional lymph node metastasis | **N0a** | one or more cytologically or histologically confirmed benign  lymph nodes |
|  |  |  | **N0b** | no radiologic or clinical evidence of locoregional lymph node metastasis |
|  | **N1a** | metastasis to level VI | metastasis to level VI, VII | |
|  | **N1b** | metastasis to level I, II, III, IV, V, VII, retropharyngeal lymph nodes | metastasis to level I, II, III, IV, V, retropharyngeal lymph nodes | |
| **Metastasis** | **M0** | no distant metastasis | The same. | |
|  | **M1** | distant metastasis | The same. | |

**AJCC TNM seventh and eighth edition: category**
