# Supplementary material for: Predictive values of the selected inflammatory index in elderly patients with papillary thyroid cancer
Source: J Transl Med. 2018 Sep 21;16:261. doi: 10.1186/s12967-018-1636-y (PMC6151008; doi:10.1186/s12967-018-1636-y)
Supplement: Supplementary file 2 — Additional file 2: Table S2. AJCC TNM seventh and eighth edition: stage. [file 12967_2018_1636_MOESM2_ESM.docx]

**AJCC TNM seventh and eighth edition: stage**

|  | **Age (Years）** | **7^th^ edition** | | | **Age**  **(Years)** | **8^th^ edition** | | |
| --- | --- | --- | --- | --- | --- | --- | --- | --- |
|  |  | **Tumor** | **Node** | **Metastasis** |  | **Tumor** | **Node** | **Metastasis** |
| **Ⅰ** | **＜45** | Any T | Any N | M0 | **＜55** | The same. | | |
| **Ⅱ** |  | Any T | Any N | M1 |  | The same. | | |
| **Ⅰ** | **≥45** | T1 | N0 | M0 | **≥55** | T1-2 | N0/NX | M0 |
| **Ⅱ** |  | T2 | N0 | M0 |  | T1-2 | N1 | M0 |
|  |  |  | | |  | T3 | Any N | M0 |
| **Ⅲ** |  | T1-3 | N1a | M0 |  | T4a | Any N | M0 |
|  |  | T3 | N0 | M0 |  |  | | |
| **Ⅳa** |  | T1-3 | N1b | M0 |  | T4b | Any N | M0 |
|  |  | T4a | N0/N1 | M0 |  |  | | |
| **Ⅳb** |  | T4b | Any N | M0 |  | Any T | Any N | M1 |
| **Ⅳc** |  | Any T | Any N | M1 |  | - - - | | |
