# Supplementary material for: Predictive values of the selected inflammatory index in elderly patients with papillary thyroid cancer
Source: J Transl Med. 2018 Sep 21;16:261. doi: 10.1186/s12967-018-1636-y (PMC6151008; doi:10.1186/s12967-018-1636-y)
Supplement: Supplementary file 3 — Additional file 3: Table S3. AUC of hematological parameters of clinicopathologic characteristics in young patients (<55 years) with PTC. [file 12967_2018_1636_MOESM3_ESM.docx]

**Supplementary table 3: AUC of hematological parameters of clinicopathologic characteristics in young patients(＜55y) with PTC**

| **Prognostic factor** | **AUC** | **95% CI** | **P** |
| --- | --- | --- | --- |
| Bilaterality |  |  |  |
| Neutrophils | 0.526 | 0.467-0.585 | 0.395 |
| Lymphocyte | 0.505 | 0.444-0.565 | 0.882 |
| NLR | 0.523 | 0.463-0.583 | 0.453 |
| LMR | 0.514 | 0.453-0.576 | 0.645 |
| MPV | 0.509 | 0.448-0.570 | 0.762 |
| PDW | 0.525 | 0.464-0.587 | 0.408 |
| Lymph node metastasis |  |  |  |
| Neutrophils | 0.506 | 0.452-0.561 | 0.820 |
| Lymphocyte | 0.523 | 0.467-0.578 | 0.422 |
| NLR | 0.524 | 0.469-0.580 | 0.389 |
| LMR | 0.568 | 0.513-0.622 | 0.017* |
| MPV | 0.512 | 0.457-0.567 | 0.668 |
| PDW | 0.505 | 0.450-0.560 | 0.874 |
| Coexistence with Hashimoto’s thyroiditis |  |  |  |
| Neutrophils | 0.571 | 0.509-0.633 | 0.020* |
| Lymphocyte | 0.588 | 0.531-0.644 | 0.004* |
| NLR | 0.500 | 0.440-0.559 | 0.987 |
| LMR | 0.510 | 0.447-0.572 | 0.744 |
| MPV | 0.516 | 0.457-0.575 | 0.594 |
| PDW | 0.501 | 0.442-0.559 | 0.983 |

Abbreviations: AUC, area under the curve of ROC; NLR, neutrophil-to-lymphocyte ratio; LMR, lymphocyte-to-monocyte ratio; MPV, mean platelet volume; PDW, platelet distribution width.

The significance level was set at P < 0.05

*Significant difference
